# Ectopic pancreatic islets in Splenic hilum and peripancreatic fat

**DOI:** 10.1186/1746-1596-3-3

**Published:** 2008-01-29

**Authors:** Kirti Gupta, RK Vasishta

**Affiliations:** 1 Department of Histopathology, Post Graduate Institute of Medical Education and Research (PGIMER), Chandigarh, India

## Abstract

The presence of pancreatic islets alone in the peripancreatic region and splenic hilum is an uncommon occurrence. Herein, we describe their presence in this rare location.

## Commentary

**Figure 1:**
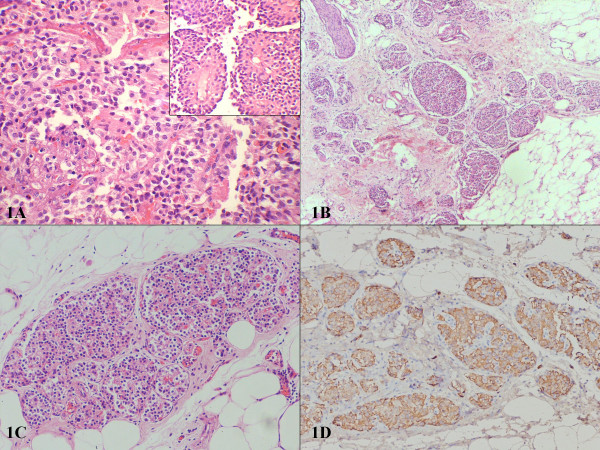
**Microphotographs of PSEN and pancreatic islets**. **a: **Within the microcystic spaces, the tumor consisted of uniformly round to cuboidal cells arranged in solid sheets and papillae (Inset), (original magnification × *200*, HE stain). **b: **Nodular aggregates of pancreatic islets randomly scattered in the fat around the splenic hilum (original magnification × *100*, HE stain). **c: **Higher magnification of islets with rounded contours (original magnification × *200*, HE stain). **d: **Beta cells present within the islets highlighted by the insulin immunostain (original magnification × *1000*, immunoperoxidase stain).

Ectopic pancreatic tissue may occur from displacement of small amounts of pancreas during embryonic development, resulting in formation of a nodule which is independent of the pancreas. It often has a proper ductal system and circulation [1,2]. In majority of the cases it is an incidental finding, less often it may be present with obstruction and ulceration. The common sites of ectopic rests are stomach, duodenum and jejunum. Rarely it may be seen in Meckel's diverticulum, umbilicus and mediastinum [3,4]. Grossly, it may be evident as firm, pale, nodular mass. Microscopically, the usual lobular architecture is maintained with variable admixture of acini, islets and ductal structures. The presence of ectopic islets without any accompanying acini or ducts is quite uncommon. Herein, we report a case of 21-year-old female with nodular mass in the tail of the pancreas. Fine needle aspiration cytology revealed a papillary lesion with features consistent with papillary-solid-epithelial neoplasm (PSEN). Subsequently excision of the mass in distal pancreas with splenectomy was done. Grossly, the mass measured 8.5 × 8 × 6 cm with circumscribed margins well distinct from the surrounding pancreatic tissue. Spleen on gross examination was within normal limits. The cut surface of the nodular mass showed variable admixture of solid and cystic areas with fine papillary excrescences. Microscopically, it revealed features of papillary solid epithelial neoplasm (Fig 1a) with presence of pseudopapillae (Fig 1a, inset) and collections of foam cells and cholesterol clefts. Random sections taken from the splenic hilum and peripancreatic fat revealed presence of ectopic islets without any accompanying acini and ducts (Fig 1b). These islets were round to ovoid with regular contours (Fig 1c). Insulin immunohistochemistry highlighted the presence of insulin-secreting beta cells within it (Fig 1d).

Rare case reports of pancreatitis occurring in the ectopic pancreatic tissue in the mesentry of the small intestine of a child have been described in the literature [5]. Ectopic islets sometimes may be symptomatic on account of production and secretion of insulin. Asymptomatic occurrence of islets is also common, however presence of islets alone without the accompanying ductal system in splenic hilum has not been previously described to the best of our knowledge. The pathologists must be aware of this rare site for correct interpretation and diagnosis.
